# Low-coverage whole genome sequencing of cell-free DNA to predict and track immunotherapy response in advanced non-small cell lung cancer

**DOI:** 10.1186/s13046-025-03348-0

**Published:** 2025-03-08

**Authors:** Florian Janke, Mateo Gasser, Arlou K. Angeles, Anja L. Riediger, Magdalena Görtz, Louise Appenheimer, Astrid K. Laut, Simon Ogrodnik, Sabrina Gerhardt, Albrecht Stenzinger, Marc A. Schneider, Michael Thomas, Petros Christopoulos, Holger Sültmann

**Affiliations:** 1 Division of Cancer Genome Research, German Cancer Research Center, Im Neuenheimer Feld 460, 69120 Heidelberg, Germany; 2 Translational Lung Research Center (TLRC), Member of the German Center for Lung Research (DZL), Heidelberg, Germany; 3 National Center for Tumor Diseases (NCT), Heidelberg, Germany; 4 Junior Clinical Cooperation Unit, Multiparametric Methods for Early Detection of Prostate Cancer, German Cancer Research Center (DKFZ), Im Neuenheimer Feld 420, 69120 Heidelberg, Germany; 5 Department of Urology, Heidelberg University Hospital, Heidelberg, Germany; 6 Faculty of Biosciences, Heidelberg University, Heidelberg, Germany; 7 Institute of Pathology, Heidelberg University Hospital, Im Neuenheimer Feld 224, 69120 Heidelberg, Germany; 8 German Cancer Consortium (DKTK), Heidelberg, Germany; 9 Translational Research Unit, Thoraxklinik at Heidelberg University Hospital, Röntgenstraße 1, 69126 Heidelberg, Germany; 10 Department of Oncology, Thoraxklinik at Heidelberg University Hospital, Heidelberg, Germany

**Keywords:** Liquid biopsy, Non-small cell lung cancer, Immunotherapy, Response prediction, Copy number variations, CfDNA fragmentation, Low-coverage whole genome sequencing

## Abstract

**Background:**

Outcomes under anti-PD-(L)1 therapy have been variable in advanced non-small cell lung cancer (NSCLC) without reliable predictive biomarkers so far. Targeted next-generation sequencing (NGS) of circulating tumor DNA (ctDNA) has demonstrated potential clinical utility to support clinical decisions, but requires prior tumor genetic profiling for proper interpretation, and wide adoption remains limited due to high costs.

**Methods:**

Tumor-agnostic low-coverage ctDNA whole genome sequencing (lcWGS) was used to longitudinally track genome-wide copy number variations (CNVs) and fragmentation features in advanced NSCLC patients (*n* = 118 samples from 49 patients) and healthy controls (*n* = 57). Tumor PD-L1 expression was available for comparison.

**Findings:**

Fragmentation features and CNVs were complementary indicators, whose combination significantly increased ctDNA detection compared to single-marker assessments (+ 20.3% compared to CNV analysis alone). Baseline fragment length alterations, but not CNVs, were significantly associated with subsequent progression-free survival (PFS; hazard ratio [HR] = 4.10, *p* = 6.58e-05) and could improve PFS predictions based on tumor PD-L1 expression alone (HR = 2.70, *p* = 0.019). Residual CNVs or aberrant fragmentation of ctDNA under ongoing therapy could stratify patients according to the subsequent response duration (median 5.8 *vs.* 47.0 months, *p* = 1.13e-06). The integrative analysis of ctDNA fragment characteristics at baseline, tumor PD-L1 expression, and residual ctDNA under ongoing treatment constituted the strongest independent predictor of PFS (*p* = 6.25e-05) and overall survival (*p* = 1.3e-03) in multivariable analyses along with other clinicopathologic variables.

**Interpretation:**

This study demonstrates the feasibility and potential clinical utility of lcWGS for the tumor-agnostic stratification and monitoring of advanced NSCLC under PD-(L)1 blockade based on CNV and fragmentomic profiling.

**Supplementary Information:**

The online version contains supplementary material available at 10.1186/s13046-025-03348-0.

## Background

Immune checkpoint blockers (ICB), in particular inhibitors of programmed death-(ligand)-1 (PD-(L)1), are routinely used in the treatment of advanced non-small cell lung cancers (NSCLC) and significantly improve patient prognosis [1, 2]. However, primary resistance to anti-PD-(L)1 therapy is common and reliable biomarkers predicting treatment efficacy are urgently needed. Several parameters – such as tumor PD-L1 expression [3], tumor mutational burden [4, 5], or microsatellite instability [6] – have been proposed, but the identification of responders remains incomplete and requires access to tumor tissue [7, 8]. Timely detection of response to ICB could drastically improve patient management by maintaining therapy for those likely to benefit from ICB, while sparing others from unnecessary treatment.

The evaluation of circulating tumor DNA (ctDNA) in plasma has emerged as a promising biomarker for treatment response prediction and monitoring across various therapeutic modalities, including ICB [9–11]. Previous studies demonstrated encouraging results, highlighting the association between on-treatment ctDNA reductions and favorable patient prognosis [7, 9, 12–15]. However, most of these studies utilized personalized ctDNA assays that require prior knowledge of genomic alterations in the tumor tissue. In advanced cancers, sufficient tumor material is not always available, posing the need for tumor-agnostic ctDNA quantification assays. Low-coverage whole genome sequencing (lcWGS) of plasma cell-free DNA (cfDNA) allows the detection of somatic copy number variations (CNVs) without prior tumor information. We and others demonstrated the suitability of lcWGS for therapy monitoring and response evaluation, particularly in advanced cancer patients who are often characterized by gross chromosomal instability [16–20]. More recently, several cfDNA fragmentation features were identified from lcWGS and linked to the abundance of ctDNA in plasma. These include the preponderance of short cfDNA fragments in cancer patients [20, 21], diverse fragment end motifs [22, 23], as well as aberrant fragment end positions when compared to cfDNA of non-cancer controls [24, 25]. The combined evaluation of CNVs and fragmentation features in cfDNA was demonstrated to enhance the sensitivity of tumor-agnostic ctDNA quantification [23] and may improve response evaluation in NSCLC patients receiving PD-(L)1 blockade.

Here, we performed a joint analysis of CNVs and fragmentation features in cfDNA of 49 advanced NSCLC patients using samples before and after 4 cycles of anti-PD-(L)1 therapy to explore the potential clinical utility of these markers.

## Methods

### Patients

118 plasma specimens were collected from 49 advanced NSCLC patients receiving anti-PD-(L)1 blockade alone or in combination with chemotherapy at the Thoraxklinik Heidelberg between September 2017 and February 2024. The collection of the cohort was focused on patients with durable and short response to ICB, potentially facilitating the identification of novel predictive markers. Patients with targetable molecular alterations (*i.e.*, mutations in *EGFR*, *BRAF*, *MET*, *HER2* as well as *ALK*, *ROS-1*, *RET* or *NTRK* fusions) were excluded from this study. Histopathological diagnosis and molecular profiling of NSCLC tumor tissue were performed at the Institute of Pathology Heidelberg, as previously described [26]. Clinical data were collected through a review of patient records (cut-off date: February 15, 2024) and included: i) tumor histology, ii) type of treatment, iii) PD-L1 tumor proportion score (TPS), iv) *TP53* mutation status, as well as v) complete blood counts and other routine laboratory values, such as hemoglobin, serum C-reactive protein (CRP), lactate dehydrogenase (LDH), and creatinine concentration. Tumor response and date of progression were verified through re-evaluation of radiologic images, *i.e.*, chest/abdominal computed tomography and magnetic resonance imaging of the brain every 8 to 12 weeks, by the investigators according to the Response Evaluation Criteria in Solid Tumors (RECIST) version 1.1 [27]. Progression-free survival (PFS) was assessed from the date of therapy initiation to disease progression or death. Treatment efficacy was dichotomized into durable clinical response (DCR; PFS > 12 months) and short clinical response (SCR; PFS ≤ 12 months). Overall survival (OS) was determined from therapy start to the patient´s death or date of last contact. Patients without disease progression or death at the time of data cut-off were censored at the date of the last contact.

To provide a reference cohort, plasma from 35 healthy controls without known tumor disease, collected at the Heidelberg University Hospital, were used. In addition, whole genome sequencing (WGS) data of cfDNA from 22 healthy individuals were obtained from literature [28]. Patient/donor information are detailed in Tab. S1.

### Plasma sample preparation and sequencing

Per patient, two 7.5 mL K_2_EDTA tubes of whole blood were collected and subjected to plasma isolation within 1 h of venipuncture using the double-spin centrifugation method [17, 18]. On average, this resulted in 3 mL of plasma which was stored at -80 °C at the Lung Biobank Heidelberg, member of the BioMaterial Bank Heidelberg (BMBH). cfDNA extraction was performed using 400—800 µL of clarified plasma with the QIAamp MinElute ccfDNA Kit (Qiagen, Hilden, Germany). WGS libraries were prepared from 1.5—5.0 ng of cfDNA using the KAPA HyperPrep reagents (Roche Diagnostics, Mannheim, Germany) and NEBNext Multiplex Oligos for Illumina adapters (New England Biolabs, Ipswich, USA), as detailed in our previous work [17, 18]. Subsequently, libraries were amplified for 9—10 PCR cycles, pooled equimolarly and sequenced on a NovaSeq6000 instrument with S4 paired-end 100-bp flow-cells (Illumina, San Diego, USA).

### Whole genome sequencing data processing

Raw sequencing data was run through a custom pipeline (detailed in Supplementary Methods) that included adapter trimming, read alignment to GRCh38/hg38, and quality filtering. The resulting bam files were used to identify copy number profiles with WisecondorX v1.2.5 [29] (default parameters). Copy number abnormality (CPA) scores were calculated to express the extent of chromosomal instability per sample [30]. In addition, ctDNA-informed (ct)CPA scores were determined in patients with detectable CNVs at therapy baseline (Supplementary Methods). The profiling of cfDNA fragment features (but not CNVs) was focused on the mononucleosomal cfDNA peak (*i.e.*, fragments ≤ 250-bp) and included an additional fragment length-filtering step. Fragment length features were extracted using custom pysam implementations and comprised: i) the enrichment of short cfDNA fragments (*i.e.*, the proportion of fragments between 126 and 135-bp) [20, 21, 31], ii) the absolute deviation of fragments between 126 and 135-bp from the median of our healthy donor cohort, iii) the quantification of fragment end trinucleotide motif diversity scores (MDS) [22, 23], iv) the analysis of fragment end position aberrancy (via information-weighted fraction of aberrant fragments (iwFAF) scores) [24], and v) the fragment end motif-based assessment of *Alu* element hypomethylation [32]. Further details on the determination of the cfDNA fragment features and per sample information on plasma collection, library preparation, sequencing data quality, and CNV as well as fragmentation biomarkers are given in the Supplementary Methods and Tab. S2.

### Statistical analyses and data visualization

Differences in (ct)CPA scores, fragment length proportions, MDS values, iwFAF scores, and *Alu* element hypomethylation were examined using the Mann–Whitney U and Wilcoxon´s paired test for independent and paired data, respectively. Correlation coefficients were calculated using Spearman´s rank correlation. For survival analyses, Kaplan–Meier curves were compared using the two-sided log-rank test and univariate/multivariable survival analyses were carried out by Cox proportional hazard models. *P*-values < 0.05 were considered significant, if not stated otherwise. All statistical analyses were performed with R v4.2.0 (R Foundation for Statistical Computing, Vienna, Austria) and relevant plots were generated using R´s ggplot2 package [33].

## Results

### Patient characteristics

A total of 49 advanced NSCLC patients were included in this study. The median age was 65 years (range 48—83) with 71.4% male patients (Table 1). The most common histological subtype was lung adenocarcinoma (75.5%), followed by lung squamous cell carcinoma (22.4%), and one patient with NSCLC not otherwise specified (NOS; 2.0%). 85.7% and 49.0% of patients had a PD-L1 TPS ≥ 1% or ≥ 50%, respectively. The *TP53* mutation status of 38/49 patients were available from a previous study [34], of which 44.7% carried a mutation. All reported *TP53* mutations were classified as pathogenic. Selected routine laboratory values (*e.g.*, complete blood counts and serum LDH) were available for all patients (Tab. S1). Anti-PD-(L)1 monotherapy (*i.e.*, pembrolizumab, nivolumab, durvalumab, or atezolizumab) was administered to 53.1% of the study population, while the remaining patients received pembrolizumab combined with carboplatin-based chemotherapy. Carboplatin was combined with (nab-)paclitaxel for squamous cell carcinomas and pemetrexed for the others. All patients were treatment-naïve for their current disease. Stage III patients received durvalumab as consolidation therapy following chemoradiotherapy. 27 patients had SCR with PFS ≤ 12 months (median = 6.8, range 3.9—9.7 months), while the other 22 comprised the DCR group with a median PFS of 56 months (range 15.6—63.2 months). In total, 118 plasma specimens were collected throughout the entire study. These included 48 pre-treatment samples, 46 samples taken after 4 therapy cycles, and 24 samples collected at disease progression (Fig. S1 and Tab. S2). All samples were subjected to lcWGS and sequenced to a median genome coverage of 2.8x (range 0.8—6.0; Tab. S2).

**Table 1:** Characteristics of study patients

NSCLC patients (*n* = 49; *n* = 118 plasma specimens)		
Age in years, median (range)		65 (48—83)
Sex, number of patients	Female	14 (28.6%)
	Male	35 (71.4%)
Stage, number of patients	III	4 (8.2%)
	IV	45 (91.8%)
Tumor histology, number of patients	Lung adenocarcinoma	37 (75.5%)
	Lung squamous cell carcinoma	11 (22.4%)
	NOS	1 (2.0%)
Type of immune checkpoint blockade, number of patients	Atezolizumab	3 (6.1%)
	Durvalumab	4 (8.2%)
	Nivolumab	4 (8.2%)
	Pembrolizumab	15 (30.6%)
	Pembrolizumab + CTx	23 (46.9%)
PD-L1 TPS, number of patients	< 1%	7 (14.3%)
	1—49%	18 (36.7%)
	≥ 50%	24 (49.0%)
*TP53* status, number of patients	Wild-type	21 (42.9%)
	Mutated	17 (34.7%)
	Unknown	11 (22.4%)
Progression-free survival in months, median (range)		9.1 (3.9—63.2)
Overall survival in months, median (range)		35.6 (5.2—64.2)

*CTx* chemotherapy, *NOS* not otherwise specified, *PD-L1* programmed death-ligand 1, *TPS* tumor proportion score

### NSCLC plasma samples are characterized by CNVs and altered cfDNA fragmentation features

**Fig. 1:**
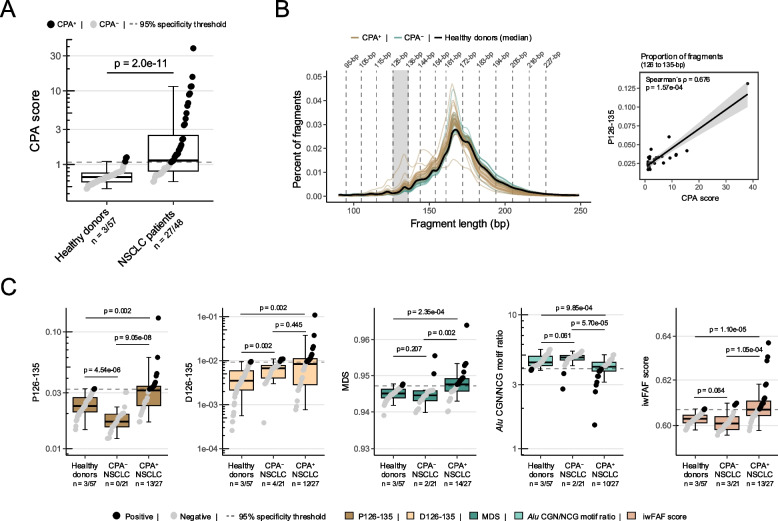
**A** Copy number abnormality (CPA) scores in healthy donors compared to pre-treatment NSCLC patients. The number of samples surpassing the 95% specificity threshold and the total number of samples per group are given below the graph. **B** Fragment length distribution of CPA^+^ (*n* = 27), CPA^–^ pre-treatment NSCLC samples (*n* = 21), and the median size profile of all healthy donors (*n* = 57). 10-bp periodicity segments are indicated by the broken vertical lines. The correlation between the proportion of fragments between 126 and 135-bp (P126-135; shaded area) and CPA scores is highlighted on the right. **C** Comparison of fragmentation features (*i.e.*, P126-135, absolute P126-135 deviation from the median of healthy donors [D126-135], motif diversity scores (MDS), *Alu* element methylation based on CGN/NCG end motif ratios, and information-weighted fraction of aberrant fragments (iwFAF) scores) between healthy donors, CPA^+^ and CPA^–^ pre-treatment NSCLC samples

CNV-based ctDNA burden (assessed by CPA scores based on lcWGS data) was significantly increased in patients compared to healthy donors (Mann–Whitney U, *p* = 2.0e-11; Fig. 1A). At 95% specificity, 27/48 (56.3%) of pre-treatment NSCLC samples were detected by the CPA scores (CPA^+^). In addition, short fragments were enriched in CPA^+^ samples compared to healthy donors (Fig. 1B). In line with a previous report [31], we observed a strong correlation between the proportion of fragments ranging from 126 to 135-bp (P126-135) and CPA scores in CPA^+^ samples (Spearman´s *ρ* = 0.676, *p* = 1.57e-04; Fig. 1B), suggesting an association between fragments of this length and ctDNA burden. In contrast, no correlation was noted between CPA scores and P126-135 in CPA^−^ and healthy donor samples (Tab. S3). While P126-135 was elevated in CPA^+^ samples (Mann–Whitney U, *p* = 0.002; Fig. 1C), we found a significant reduction of P126-135 in CPA^−^ samples (Mann–Whitney U, *p* = 4.54e-06) when contrasted with our healthy donor cohort. As a consequence, we computed the absolute P126-135 deviation from the median P126-135 of our healthy donors (D126-135) to quantify fragment length changes into any direction. In relation to the healthy donors, D126-135 was significantly altered in both CPA^+^ and CPA^−^ samples (Fig. 1C). Next, we assessed 3 additional cfDNA fragmentation features based on fragment end trinucleotides (*i.e.*, MDS and *Alu* element CGN/NCG ratios) and fragment end positions (*i.e.*, iwFAF scores). These metrics were previously established as cancer-associated markers [22, 24, 32]. In our cohort, they showed significant alterations in CPA^+^ samples compared to healthy donors (Fig. 1C), highlighting their association with ctDNA.

Subsequently, we computed pair-wise correlations of the cfDNA biomarkers in all pre-treatment patient and healthy donor samples, separately. The majority of comparisons in NSCLC patients demonstrated moderate (Spearman´s |ρ|≥ 0.25, *p* < 0.05) and strong (Spearman´s |ρ|≥ 0.7, *p* < 0.05) correlations (Fig. S2A), while most correlations were reduced (or absent) in healthy donors (Fig. S2B). Of interest, some fragmentation features remained significantly correlated in healthy individuals (*e.g.*, MDS *vs.* P126-135), indicating that those features might be influenced by other, non-malignant factors. In patient samples, MDS and *Alu* element CGN/NCG ratios (among others), additionally, correlated with pre-treatment platelet counts and other laboratory values (Fig. S2C), a phenomenon also persistent in CPA^−^ samples, highlighting the correlation´s independence from ctDNA burden (Fig. S2D). Other physiological and clinical factors (*e.g.*, age, sex, PD-L1 TPS or *TP53* mutation status; Fig. S2C, E and F) were not correlated to any of the cfDNA biomarkers. Furthermore, the presence of brain metastases was not associated with reduced cfDNA biomarker levels, as these mostly co-occurred with additional metastases in other organs (Fig. S2G).

### Fragment features complement CNVs for the detection of ctDNA

**Fig. 2:**
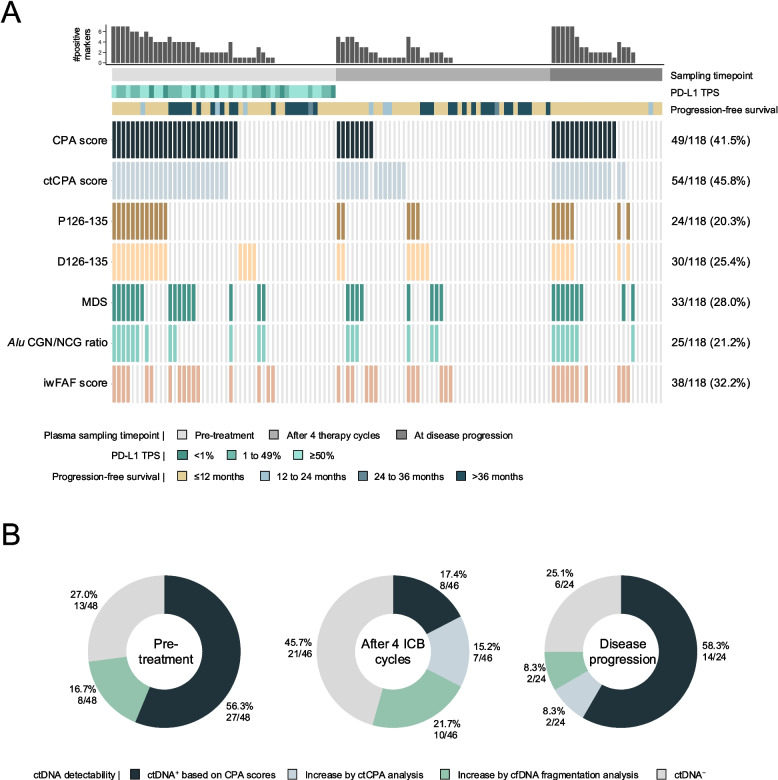
**A** Oncoprint highlighting the detectability of circulating tumor DNA (ctDNA) based on cell-free copy number variations (CNVs; CPA and ctCPA score), cell-free DNA (cfDNA) fragment length (P126-135 and D126-135), fragment end motif diversity scores (MDS), fragment end-based DNA methylation inference (*Alu* CGN/NCG ratio), and fragment end position (iwFAF score). Colored tiles indicate detectable ctDNA at 95% specificity assessing the respective cfDNA biomarker. The absolute and relative number of ctDNA^+^ samples is given on the right side of the plot. **B** ctDNA detectability per sampling timepoint, illustrating the added value of joint CNV and cfDNA fragmentation analysis. Colors indicate the evaluation of CPA scores alone, the number of additional ctDNA^+^ samples when ctCPA scores were analyzed in addition to CPA scores, and when cfDNA fragmentation was assessed in addition to CPA and ctCPA scores. cfDNA fragmentation-positivity refers to the detectability of at least one of the 5 evaluated features. CPA, copy number abnormality; ctCPA, ctDNA-informed CPA; iwFAF, information-weighted fraction of aberrant fragments; PD-L1, programmed death-ligand 1; TPS, tumor proportion score

Minimally invasive ctDNA detection has many potential applications in cancer care, however, the limited sensitivity of most assays impedes clinical implementation. We tested if the joint evaluation of CNVs and cfDNA fragmentation features, from the same lcWGS dataset, could improve ctDNA detection. To enhance the CNV-based ctDNA assessment in longitudinally collected plasma, we established the ctCPA score that expressed the deviation from copy number neutrality at patient-specific CNVs found in previous plasma samples of the same patient (Supplementary Methods; [19]). Hereby, we aimed to reduce background noise similar to tumor tissue-informed ctDNA evaluation assays [35, 36]. At 95% specificity, CPA scores detected 49/118 (41.5%) of NSCLC patient samples and 54/118 (45.8%) samples were ctCPA^+^ (Fig. 2A). In 4 instances, CPA, but not ctCPA scores, detected tumor signals. These cases had CPA scores marginally above the detectability threshold without obvious CNVs, therefore prohibiting the calculation of ctCPA scores. Fragment length metrics (*i.e.*, P126-135 and D126-135) identified cancer signals in 24/118 (20.3%) and 30/118 (25.4%) samples, respectively. MDS, *Alu* element CGN/NCG motif ratios, and iwFAF scores detected ctDNA in 33/118 (28.0%), 25/118 (21.2%) and 28/118 (32.2%) samples, respectively. In total, cancer was detected in 78/118 (66.1%; at 95% specificity) samples by at least one of the metrics. When compared to the evaluation of CPA scores alone, the combination of all markers identified 29 additional samples and 8 patients. Interestingly, we found that cfDNA biomarker integration demonstrated the largest improvement for samples taken after 4 ICB cycles (Fig. 2B). Here, the joint analysis of CPA, ctCPA scores, and cfDNA fragmentation detected ctDNA in 25/46 (54.3%) of patients, demonstrating a substantial increase compared to the evaluation of CPA scores alone (8/46 [17.4%]). An overview of ctDNA detectability by plasma collection timepoint and the total number of ctDNA^+^ patients for each biomarker and their integration is given in Tab. S4. Furthermore, we observed significantly higher D126-135 values in SCR *versus* DCR patients at baseline (Mann–Whitney U, *p* = 0.006), while CPA, P126-135, MDS, and iwFAF scores after 4 ICB cycles were significantly increased in the SCR group (Fig. S3). These results highlight their different informative values at the various plasma collection timepoints.

### cfDNA fragment length at baseline predicts response to ICB and complements PD-L1 TPS

**Fig. 3:**
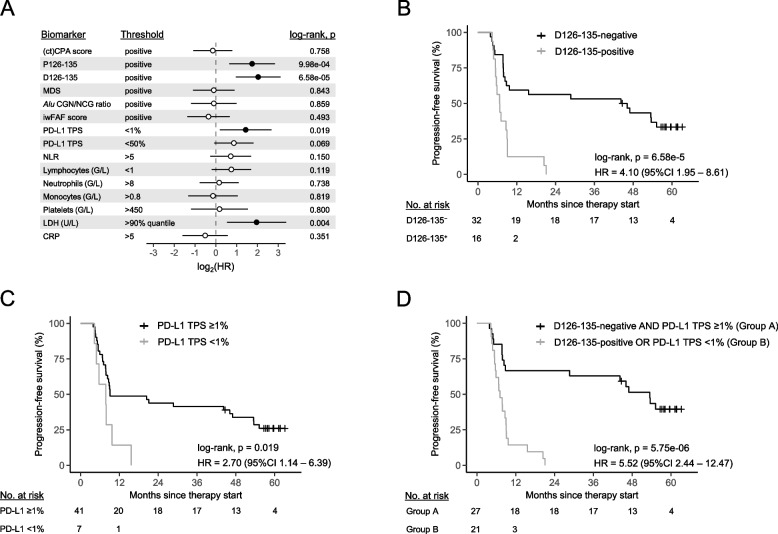
**A** Forest plot reporting the hazard ratios (HRs) of disease progression and their 95% confidence intervals for cfDNA biomarkers at baseline, clinically relevant PD-L1 tumor proportion scores (TPS), and different routine laboratory values. Statistically significant results are indicated by filled dots. Threshold to dichotomize patients are given for each biomarker. ‘Positive’ refers to detectable levels of the respective biomarker according to its 95% specificity threshold. Hemoglobin and creatinine concentrations are not illustrated due to group sizes < 4. Progression-free survival (PFS) according to the detectability (at 95% specificity) of D126-135 (**B**) and PD-L1 tumor proportion scores (TPS) ≥ 1% (**C**) at anti-PD-(L)1 therapy baseline. **D** Association between PFS and the combination of D126-135 detectability and/or PD-L1 TPS < 1% at therapy baseline. Groups were compared by two-sided log-rank tests and hazard ratios were calculated via univariate Cox proportional hazard models. CRP, C-reactive protein; D126-135, deviation of fragments between 126 and 135-bp from healthy donors; iwFAF, information-weighted fraction of aberrant fragments; LDH, lactate dehydrogenase; MDS, motif diversity score; NLR, neutrophil-to-lymphocyte ratio; P126-135, proportion of fragments between 126 and 135-bp; PD-L1, programmed death-ligand 1

Here, we assessed the predictive value of our cfDNA biomarkers at therapy baseline (*i.e.*, CNVs and fragmentation features) alongside tumor tissue PD-L1 TPS, neutrophil-to-lymphocyte ratios (NLR), data from complete blood counts, and various clinical blood values (Supplementary Methods). Patient groups were split at the cfDNA biomarker´s respective detectability threshold (at 95% specificity; cfDNA biomarkers), established physiological levels (routine laboratory values; Supplementary Methods), or clinically relevant thresholds (≥ 1% and ≥ 50% PD-L1 TPS). In addition, increasing quantiles (*i.e.*, 25%, 50%, 75%, and 90%) of the cohort´s pre-treatment samples were used to dichotomize patients. CPA and ctCPA scores were combined to identify detectable CNVs in samples that exceeded the 95% specificity threshold in at least one of the markers. When examining the relationship between the cfDNA biomarkers and PFS, we observed significant associations between detectable P126-135 (hazard ratio [HR] = 3.35, 95% confidence interval [CI] 1.56—7.16; *p* = 9.98e-04) and D126-135 (HR = 4.10, 1.95—8.61 95%CI; *p* = 6.58e-05) levels and short therapy response (Fig. 3A, B and Fig. S4A). Notably, several of the evaluated quantile thresholds for P126-135 and D126-135 also correlated with short PFS (Tab. S5). At ICB baseline, none of the other cfDNA biomarkers was significantly associated with therapy response (Fig. 3A and Tab. S5). Furthermore, PD-L1 expression ≥ 1% correlated with favorable PFS (HR = 2.70, 95%CI 1.14—6.39; *p* = 0.019; Fig. 3C), while high LDH concentration (> 90% quantile; 371.9 U/L) predicted early disease progression (HR = 3.89, 95%CI 1.45—10.38; *p* = 0.004; Fig. S4B). Detectable D126-135 levels, PD-L1 TPS ≥ 1%, and high LDH concentrations remained independently predictive in a multivariable Cox regression analysis (MVA), adjusting for age, gender, tumor histology, and therapy type (Tab. S6). Importantly, the integration of PD-L1 TPS and D126-135 detectability improved the identification of patients with short PFS (HR = 5.52, 95%CI 2.44—12.37; *p* = 5.75e-06; Fig. 3D) compared to single-markers. The biomarker combination remained independently significant in a MVA (Tab. S6). In an OS analysis, D126-135 (> 95% specificity cut; HR = 2.31, 95%CI 1.13—4.72; *p* = 0.018) and LDH (> 90% quantile; HR = 4.32, 95%CI 1.57 – 11.90; *p* = 0.002) demonstrated a significant relation to short survival (Fig. S5A, B and Tab. S5) in univariate but not MVA.

### Residual CNVs and cfDNA fragmentation features after 4 cycles of anti-PD-(L)1 therapy predict therapy outcome

**Fig. 4:**
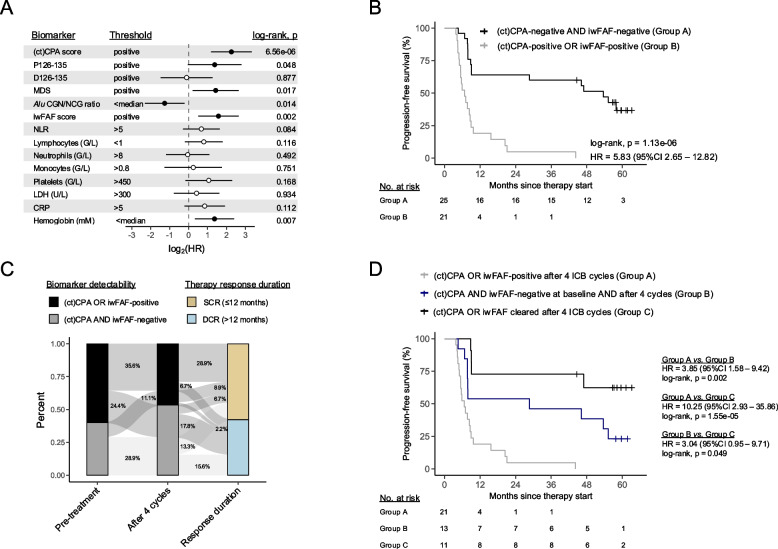
**A** Forest plot of progression-free survival (PFS) univariate Cox regression for cfDNA biomarkers and routine laboratory values measured after 4 therapy cycles. Filled dots represent significant results. Patients were grouped based on the indicated thresholds. ‘Positive’ refers to biomarkers exceeding its respective 95% specificity threshold. Comparisons with group sizes < 4 are not included. **B** Association between PFS and combined evaluation of (ct)CPA and iwFAF scores detectability after therapy cycle 4. **C** Distribution of patients with and without residual ctDNA (evaluated via (ct)CPA and iwFAF score detecability) before and after 4 therapy cycles, showing the association of biomarkers status (*i.e.*, detectable *vs.* undetectable) and duration of response. **D** Kaplan–Meier curve comparing PFS in patients with undetectable (ct)CPA and iwFAF scores at baseline and after 4 therapy cycles to patients that clear marker detectability and patients that remain biomarker positive at follow-up. Groups were compared by two-sided log-rank tests and hazard ratios (HR) were calculated via univariate Cox proportional hazard models. CNV, copy number variation; CRP, C-reactive protein; ctCPA, ctDNA-informed copy number abnormality; ctDNA, circulating tumor DNA; DCR, durable clinical response; iwFAF, information-weighted fraction of aberrant fragments; LDH, lactate dehydrogenase; MDS, motif diversity score; NLR, neutrophil-to-lymphocyte ratio; SCR, short clinical response

We next focused on the association of cfDNA biomarker abundances after 4 ICB cycles and therapy response. When considering individual biomarkers, residual CNVs (*i.e.*, CPA and/or ctCPA scores > 95% specificity) demonstrated the strongest association with short time to disease progression (HR = 4.78, 95%CI 2.27—10.08; *p* = 6.56e-06; Fig. 4A and Fig. S4C). Additionally, detectable iwFAF scores, MDS, P126-135 as well as above-median *Alu* CGN/NCG ratios were significantly correlated with adverse PFS (Fig. 4A and Fig. S4D-G). Of the evaluated blood values, only hemoglobin concentrations above the cohort´s median were significantly associated with favorable PFS (Fig. S4H). The integration of two or more cfDNA biomarkers resulted in improved PFS prediction for various marker-combinations (Tab. S5). Here, the conjunction of residual CNVs and/or detectable iwFAF scores (hereafter denoted as ‘residual ctDNA’) after 4 ICB cycles performed best in predicting early disease relapse (HR = 5.83, 95%CI 2.65—12.82; *p* = 1.13e-06; Fig. 4B). In MVA analyses – adjusting for age, gender, tumor histology and therapy type – residual CNVs, above-median hemoglobin concentrations, and residual ctDNA remained independently significant (Tab. S6). Residual ctDNA was also correlated with short OS (HR = 2.95, 95%CI 1.41—6.16; *p* = 0.003; Fig. S5C).

Despite the good predictive performance of various cfDNA biomarkers, we observed several patients with short (≤ 12 months) PFS irrespective of undetectable marker abundances. Patients with undetectable marker levels at therapy baseline and after 4 ICB cycles (double-negative) were almost evenly distributed between the SCR (6/13 [46.2%]) and DCR groups (7/13 [53.8%]; Fig. 4C). By contrast, patients with biomarker clearance (*i.e.*, positive at baseline and negative after 4 ICB cycles) predominantly belonged to the DCR group (8/11 [72.7%]), while residual ctDNA after 4 ICB cycles were mainly found in patients with SCR (17/21 [80.9%]). The lower predictive value of undetectable biomarker levels after 4 ICB cycles in double-negative cases was further corroborated by the significantly shorter PFS compared to patients with biomarker clearance (HR = 3.04, 95%CI 0.95—9.71; *p* = 0.049; Fig. 4D).

### Dynamic CPA scores reflect disease kinetics under anti-PD-(L)1 therapy

Next, we evaluated whether cell-free CNVs and/or cfDNA fragmentation features could dynamically track disease kinetics in NSCLC patients under ICB treatment. To this end, we first investigated biomarker abundance changes between therapy baseline and samples taken after 4 ICB cycles in DCR and SCR patients, separately. Reflecting the sustained therapeutic response of the DCR group, CPA, ctCPA and MDS scores were significantly reduced after 4 ICB cycles when compared to the corresponding pre-treatment sample (Fig. S6). Considering the same sampling timepoints in SCR patients, none of the investigated cfDNA biomarkers demonstrated a significant change in any direction. This likely reflected the varying response duration after the administration of the 4th ICB cycle in the SCR group, ranging from 5 days to 6.4 months (median = 3.0 months). In contrast, the earliest relapse in the DCR group was noted one year after ICB cycle 4 was given (median = 52.2 months, range 12.0—59.9 months). When focusing on biomarker kinetics from ICB cycle 4 to the timepoint closest to disease progression (median = 26 days, range = 1—118 days), we observed significant CPA, ctCPA, and P126-135 increases and a significant *Alu* CGN/NCG ratio decrease leading to disease progression (Fig. S6). Taken together, only ctCPA and CPA scores consistently reflected both therapy response and failure in our cohort. This finding concurred with the previously described association between PFS and residual CNVs.

### A combination of cfDNA fragment length, PD-L1 TPS, and residual ctDNA predicts therapy outcome

**Fig. 5:**
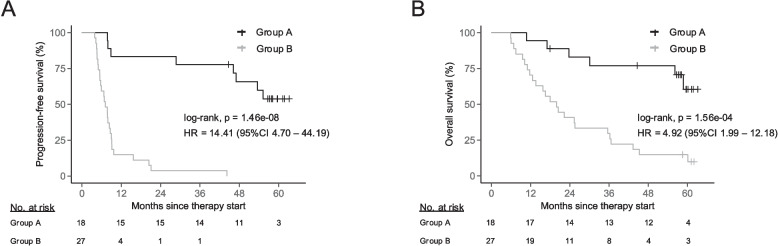
Comparison of progression-free (PFS; **A**) and overall survival (OS, **B**) between patient groups separated according to the following criteria: Group **A**; PD-L1 tumor proportion score (TPS) ≥ 1%, undetectable baseline D126-135 levels, as well as undetectable (ct)CPA and iwFAF scores after 4 therapy cycles. Group **B**; PD-L1 TPS < 1% or marker detectability for at least one of the evaluated cfDNA biomarkers. cfDNA biomarker detectability was assessed using the markers respective 95% specificity threshold. Groups were compared using two-sided log-rank tests, and hazard ratios (HR) were determined through univariate Cox proportional hazards models. cfDNA, cell-free DNA; ctCPA, ctDNA-informed copy number abnormality; D126-135, deviation of fragments between 126 and 135-bp from healthy donors; iwFAF, information-weighted fraction of aberrant fragments; PD-L1, programmed death-ligand 1

Finally, we assessed whether the combined evaluation of cfDNA biomarkers at ICB baseline and after 4 therapy cycles could enhance the prediction of therapy success. To this end, we merged information from both sampling timepoints and stratified patients into two groups: Group A included those with PD-L1 TPS ≥ 1%, undetectable pre-treatment D126-135 levels, and no residual ctDNA after 4 therapy cycles. Group B included patients that were positive for at least one of the above-mentioned cfDNA biomarkers or demonstrated a PD-L1 TPS < 1%. We observed significantly shorter PFS (HR = 14.41, 95%CI 4.70—44.19; *p* = 1.46e-08; Fig. 5A) and OS in patients of Group B (HR = 4.92, 95%CI 1.99—12.18; *p* = 1.56e-04; Fig. 5B), which remained independently significant in MVAs (Tab. S6). Compared to the analysis of individual biomarkers or single sampling timepoint, the combined approach was most effective in identifying patients with dismal PFS and OS. Notably, PFS and OS prediction remained significant when PD-L1 TPS was excluded from the analysis, highlighting the predictive value of cfDNA analysis without information from the patient´s tumor tissue (Fig. S4I and Fig. S5D).

## Discussion

The identification of NSCLC patients with sustained response to anti-PD-(L)1 therapy remains challenging. To date, only few predictive biomarkers found clinical implementation; however, their performance varies considerably between patients and cancer types [1, 2]. Recently, on-treatment ctDNA kinetics were demonstrated to correlate with therapy outcome in NSCLC and other cancer entities [7, 9, 12–15]. In this study, we systematically evaluated the predictive value of both cell-free CNVs and cfDNA fragmentation features in NSCLC patients before and during anti-PD-(L1) blockade. We found that the integration of global chromosomal instability, aberrant fragment end positions, and altered fragment lengths – derived from the same lcWGS dataset – are complementary in predicting therapy success and outperformed response prediction based on PD-L1 TPS. These findings were independent of tumor histology and ICB modality. In particular, the predictive value of pre-treatment fragment length alterations has not been described previously. Furthermore, we demonstrated that the integrative analysis of PD-L1 TPS and cfDNA biomarkers enhanced response prediction and might advance guidance of ICB therapy.

A growing number of studies support the role of on-treatment ctDNA quantification for the early identification of response to anti-PD-(L)1 therapy [7, 9, 12–15]. The majority of these reports used personalized assays to assess ctDNA levels. As a consequence, patients without available tumor tissue were excluded from the response evaluation. Tumor-agnostic methodologies – such as lcWGS – could expand ctDNA-driven response assessment to more patients and reduce delays in obtaining results. Particularly for patients with advanced tumors, lcWGS could represent a valuable alternative to personalized assays, since tissue biopsies are not always available and timely treatment initiation is crucial. Furthermore, the low cfDNA input requirements and low sequencing costs of lcWGS highlight its universal applicability even in resource-restrained settings [16]. Numerous studies showcased the information-density of lcWGS, allowing the quantification of genome-scale chromosomal instability alongside various cfDNA fragmentation features [16–18, 20–25, 28, 30, 32]. However, no study has yet assessed the (joint) value of these biomarkers for the projection of ICB treatment success in NSCLC. We determined cfDNA fragment length alterations (*i.e.*, P126-135 and D126-135), fragment end motif diversities/ratios (*i.e.*, MDS and *Alu* CGN/NCG ratios), and aberrations in their positions (*i.e.*, iwFAF scores) from lcWGS data and associated these features to ICB response. In addition, we derived a measure for global chromosomal instability (*i.e.*, CPA score) and refined CNV assessment in longitudinal samples by integrating information of previous CNV^+^ samples to reduce background noise and increase sensitivity (*i.e.*, ctCPA score [19]). ctCPA scores were instrumental in identifying residual disease after 4 therapy cycles, detecting CNVs in 7 CPA^−^ patients.

For the first time, we described that cfDNA fragmentation features and CNVs offer differential yet complementary predictive value at baseline and during anti-PD-(L)1 therapy. In our study, pre-treatment fragment length alterations (*i.e.*, P126-135 and D126-135) identified patients with short duration of response. Notably, the predictive value of D126-135 was superior to PD-L1 TPS assessment and further improved when both markers were combined. While some studies have reported weak or no associations between baseline ctDNA abundance and ICB outcome [9, 15], others have suggested that baseline ctDNA levels may serve as a biomarker for predicting ICB response in NSCLC patients [37, 38]. These contrasting findings highlight the ongoing debate in the field and emphasize the potential added value of pre-treatment fragment length evaluations. While validation in an independent cohort is needed, these findings could help to identify refractory patients even before therapy initiation. On-treatment ctDNA kinetics have been consistently shown to correlate with therapy outcomes in previous studies [7, 9, 12–15]. Accordingly, we observed that the abundance of various cfDNA biomarkers after 4 ICB cycles identified patients prone to early treatment failure. Residual ctDNA, as assessed by (ct)CPA and/or iwFAF score detectability after 4 therapy cycles, demonstrated the strongest predictor of adverse therapy outcome. Interestingly, residual ctDNA remained predictive irrespective of ctDNA abundance at baseline, while undetectable ctDNA levels identified durable responders only in the case of ctDNA clearance after baseline ctDNA-positivity. Consequently, follow-up ctDNA evaluations could be confined to patients with ctDNA^+^ pre-treatment samples for cost-efficiency. Alternatively, patients could be stratified into risk groups according to their baseline and follow-up ctDNA levels, with the highest risk assigned to those with residual ctDNA, followed by consistently ctDNA^−^ patients, and those who clear ctDNA. The joint analysis of pre-treatment PD-L1 TPS, D126-135, and residual ctDNA after 4 therapy cycles performed best in predicting therapy response as well as patient survival. This emphasizes the importance of multimodal biomarker assessment and their differential informational value at varying sampling timepoints. Importantly, the predictive value was only marginally reduced without PD-L1 TPS assessment, demonstrating that tumor-agnostic lcWGS alone can identify patients likely to respond to ICB.

We focused our on-treatment response evaluation on samples taken after 4 therapy cycles. So far, there is no consensus regarding the optimal timepoint to interrogate ICB success. Recent studies compared multiple sampling intervals and suggested different timepoints depending on the administered therapy [7, 9, 13–15, 39]. Pellini et al*.* recommended ctDNA evaluation at the beginning of cycle 4 in NSCLC patients receiving ICB-chemotherapy combinations [39]. Other studies measured ctDNA after 3 cycles in patients treated with pembrolizumab alone [9, 12, 15]. The implementation of an early timepoint for ctDNA-driven response evaluation should precede radiographic assessment and might lead to timely therapy adjustment. Our results demonstrate response prediction independent of histologic NSCLC subtype and therapy modality, emphasizing the robustness of the investigated timepoint. However, some patients experienced disease progression shortly after the administration of ICB cycle 4, mitigating the potential clinical impact.

The biological mechanisms behind the non-random cfDNA fragmentation patterns are not fully understood and remain subject to further research [23]. Here, we demonstrated moderate associations between fragment end motifs and markers for cell damage (*i.e.*, LDH concentration), inflammation (*i.e.*, CRP), and platelet counts. These results suggest that altered fragmentation might be additionally influenced by factors other than ctDNA burden. In this study, changes in fragment length provided insights into therapy response that could not be explained solely by ctDNA burden. Although fragment length metrics showed no association to any of the investigated blood cells/values, we cannot rule out that their predictive value represents the reflection of other influencing factors not investigated within the scope of this study.

The main limitations of this study are related to its retrospective design and relatively small patient cohort. Therefore, validation of the results and establishment of robust thresholds with adequate sensitivity and specificity for the clinical implementation of (ct)CPA score and cfDNA fragmentation features will be necessary in future prospective studies. The heterogeneity of NSCLC subtypes and ICB modalities represent another limitation, but could also be considered a strength as we identified biomarkers robust to these factors. Furthermore, we recognize that the cohort used here was not representative of a real-word NSCLC patient population. Patients with intermediate response durations (*i.e.*, 10—15 months) were not included. Therefore, the results of this report should be confirmed in a larger cohort that includes patients with intermediate PFS. Additionally, a larger cohort could facilitate the application of machine learning algorithms to improve the sensitivity of residual disease detection. Although remarkable therapy outcome stratification was achieved, we still identified patients without residual ctDNA that relapsed early. This suggests that assays with enhanced sensitivity might further improve ctDNA-based response assessments. In this regard, multimodal approaches integrating multiple ctDNA-derived analytes could improve residual ctDNA detection and response stratification. Beyond fragmentation and CNV-based analyses, tumor-specific mutations and DNA methylation patterns could aid ctDNA detection, particularly in cases with low tumor burden.

## Conclusion

This study highlights the feasibility and clinical relevance of lcWGS for tumor-agnostic response prediction and monitoring in NSCLC patients undergoing anti-PD-(L)1 therapy. We show that integrating CNV and fragmentomic features improves response stratification, outperforming PD-L1 TPS alone. Pre-treatment fragment length alterations identified patients with short response duration, while residual ctDNA after four therapy cycles was the strongest predictor of treatment failure. These findings support the use of lcWGS as a cost-efficient, broadly applicable tool for ctDNA-driven therapy guidance.

## Supplementary Information

Supplementary Material 1.

Supplementary Material 2.

## Data Availability

Plasma whole genome sequencing data that support the findings of this study are deposited in the European Genome-Phenome Archive (EGA) under accession numbers EGAS50000000441 (NSCLC patient data) EGAS50000000163 (healthy donor data). Sequencing data from Peneder et al. (10.1038/s41467-021-23445-w) was previously deposited to the EGA under accession EGAS00001005127. All source code is available from the corresponding author upon request.

## Abbreviations

cfDNA: Cell-free DNA
CI: Confidence interval
CNV: Copy number variation
CPA: Copy number abnormality
CpG: Cytosine-guanine dinucleotide
CRP: C-reactive protein
ctCPA: CtDNA-informed copy number abnormality
ctDNA: Circulating tumor DNA
CTx: Chemotherapy
D126-135: Deviation of fragments between 126 and 135-bp from healthy donors
DCR: Durable clinical response
FDR: False discovery rate
Hb: Hemoglobin
HR: Hazard ratio
ICB: Immune checkpoint blockade
iwFAF: Information-weighted fraction of aberrant fragments
LDH: Lactate dehydrogenase
MDS: Motif diversity score
NLR: Neutrophil-to-lymphocyte ratio
NOS: Not otherwise specified
NSCLC: Non-small cell lung cancer
OS: Overall survival
P126-135: Proportion of fragments between 126 and 135-bp
PD-L1: Programmed death-ligand 1
PFS: Progression-free survival
RECIST: Response Evaluation Criteria in Solid Tumors
SCR: Short clinical response
TPS: Tumor proportion score
WGS: Whole genome sequencing
