# Supplementary material for: Low-coverage whole genome sequencing of cell-free DNA to predict and track immunotherapy response in advanced non-small cell lung cancer
Source: J Exp Clin Cancer Res. 2025 Mar 8;44:87. doi: 10.1186/s13046-025-03348-0 (PMC11889826; doi:10.1186/s13046-025-03348-0)
Supplement: Supplementary file 1 — Supplementary Material 1. [file 13046_2025_3348_MOESM1_ESM.pdf]

Low-coverage whole genome sequencing of cell-free DNA to predict and track immunotherapy response in advanced non-small cell lung cancer

Florian Janke[1,2,3,*], Mateo Gasser[1,3], Angeles K. Angeles[1,2,3], Anja L. Riediger[1,3,4,5,6], Magdalena Görtz[4,5], Louise Appenheimer[1,3], Astrid K. Laut[1,3], Simon Ogrodnik[1,3], Sabrina Gerhardt[1,3], Albrecht Stenzinger[2,7,8], Marc A. Schneider[2,9], Michael Thomas[2,9,10], Petros Christopoulos[2,9,10], Holger Sültmann[1,2,3,8,*]

Table of contents

Supplementary Methods 3

Physiologic levels of blood cell count and clinical blood values 3

Whole genome sequencing data processing pipeline 3

Copy number variation analysis 4

Fragment length profiling 5

Fragment end motif analysis 5

Analysis of fragment end position aberrancy 6

Inference of *Alu* element hypomethylation from fragment end motifs 6

Supplementary Figure 1 7

Supplementary Figure 2 8

Supplementary Figure 3 9

Supplementary Figure 4 10

Supplementary Figure 5 11

Supplementary Figure 6 12

Supplementary references 14

***Note:** Supplementary tables are provided in a separate excel file.

Supplementary Methods

Physiologic levels of blood cell count and clinical blood values

To dichotomize patients based on available blood cell counts (*i.e.*, neutrophil, lymphocyte, monocyte, platelet counts) or clinical blood values (*i.e.*, LDH, CRP, hemoglobin, creatinine concentrations), established physiological levels of the respective analytes were utilized:

- Neutrophil count (G/L): >8
- Lymphocyte count (G/L): <1
- Monocyte count (G/L): >0.8
- Platelet count (G/L): >450
- NLR: >5
- LDH concentration (U/L): >300
- CRP concentration (mg/L): >5
- Hemoglobin molarity (mM): <7.5
- Creatinine concentration (mg/dL): >1.2

Whole genome sequencing data processing pipeline

Adapter sequences were trimmed from the 3´-end of the raw fastq reads using Cutadapt v3.7 (1) with default parameters. Subsequently, reads were mapped to the reference genome GRCh38/hg38 by bowtie2 v2.3.5.1 (2) in paired mode, discarding any fragment of lengths <30-bp and >700-bp. Aligned reads were sorted by chromosomal coordinates using samtools v1.9 (3) and PCR duplicates were excluded with Picard´s (v2.25.1) MarkDuplicates tool. Next, quality filtering was conducted (samtools v1.9) to retain only properly paired reads with MAPQ values >20 and sequencing data quality was assessed by fastqc v0.11.5. Due to the high sequence coverage of the data obtained from Penderer *et al.* 2021 (4) (mean genome coverage = 14.65x; **Tab. S2**), we downsampled the corresponding bam files to approximate the in-house data coverage

(median genome coverage = 2.8x) using samtools v1.9. As the fragment feature analyses were focused on the mononucleosomal cfDNA peak, an additional fragment length filtering step by samtools v1.9 was implemented that excluded fragments >250-bp. This step was omitted for the CNV analysis. A custom pysam (3, 5) implementation and the FrEIA toolkit (6) were used to extract the following features of each sequenced cfDNA fragment: i) fragment length, ii) GC content, iii) fragment end trinucleotide (*i.e.*, the first 3 bases from the 5´-end of a cfDNA fragment), and iv) the fragment´s start and end chromosomal coordinate.

Copy number variation analysis

WisecondorX v1.2.5 (7) with default parameters was used to call genome-wide CNVs at 1000-bp genomic windows. To estimate the diploid background, we employed the WGS data of our 57 healthy donor samples as a panel-of-normals. Healthy donor samples were analyzed using a panel-of-normals reduced by the currently analyzed sample. Genomic sections of estimated equal copy numbers were identified by circular binary segmentation (8). To obtain robust copy number profiles, we bootstrapped the panel-of-normals with 100 iterations and considered the most frequently called copy number state as the 'true' consensus state. By this means, we made sure that CNVs were not affected by individual reference samples within the panel-of-normals. The extent of chromosomal instability per plasma sample was numerically expressed using the CPA score (9). In this study, CPA scores were determined using the bootstrapped consensus copy number profiles. For patients with detectable CNVs, we devised a personalized ctCPA score that focused the analysis of subsequent longitudinal samples to the patient-specific CNVs found in one or multiple previous samples (10). The ctCPA score calculation is show in **eq. 1**:

$$ctCPA\ score = \sum_{i=1}^{n} (Z_i \times D_i)\ /n \tag{1}$$

Here, i represents the current and n the total number of copy number segments within a sample. Z_i and D_i denote the z-score and directionality of the ith segment, respectively. The directionality refers to the copy number state found in the previous sample(s) and can take one of three values (gain: +1; loss: –1; neutral: 0). (ct)CPA scores were calculated after exclusion of chromosomes X and Y. Scores are expressed per 100-Mb (**Tab. S2**). As ctCPA scores were patient-specific, thresholds denoting CNV-based ctDNA detectability had to be determined for each patient individually. Hence, patient-specific ctCPA scores were additionally calculated for all healthy donors to estimate background noise. To capture ctCPA score kinetics, we calculated for all longitudinal samples, including the CPA-positive sample used to derive the patient-specific CNV compendium.

Fragment length profiling

Global fragment length profiles were computed using the '*template_length'* function within a custom pysam (v0.16.01) implementation. Fragment length proportions were calculated relative to all fragments of a size ≤250-bp. To quantify fragment length changes – within a given size window – in both positive and negative directions, we calculated the absolute fragment length proportion deviation from the median proportion in our healthy donor cohort. For healthy donors the median fragment length proportion was calculated excluding the currently assessed sample.

Fragment end motif analysis

Fragment end trinucleotides were extracted with the FrEIA toolkit (6). Afterwards, batch-effects within the 64 3-mers were eliminated using the '*Combat_seq'* (11) function of the SVA R package. Batches were defined according to the center of plasma sample collection (**Tab. S2**). Motif diversity scores (MDS) (12) – denoting the normalized Shannon´s entropy of 3-mer proportions – were calculated to quantify the end motif diversity per sample. Here, high MDS values indicated increased cfDNA end motif varieties.

Analysis of fragment end position aberrancy

A map of genomic regions recurrently protected from nuclease degradation in healthy donors was retrieved from Budhraja *et al.* 2023 (13) and lifted over from GRCh37/hg19 to GRCh38/hg38 using the UCSC liftOver tool (14). cfDNA fragments with at least one end located within a protected region were deemed 'aberrant', while region-spanning fragments were considered 'non-aberrant'. Fragments without intersection with any of the protected regions were excluded from this analysis. The probability of a fragment being aberrant/non-aberrant given its size and GC content was determined using our healthy donor samples. Each fragment was weighted based on this probability to minimize GC content and length biases. The iwFAF score (*i.e.*, the number of weighted aberrant relative to the total number of weighted fragments) was calculated to quantify sample-wide fragment end position aberrancy. For the iwFAF score calculation of healthy donors, the samples used to determine weighing-probabilities were reduced by the currently assessed sample.

Inference of *Alu* element hypomethylation from fragment end motifs

The CGN/NCG end motif ratio was used to infer the methylation state of cytosine-guanine dinucleotides (CpGs). It quantifies the number of 5´-CGN (*i.e.*, CGA, CGT, CGG, or CGC) relative to the number of 5'-NCG end motifs (*i.e.*, ACG, TCG, GCG, or CGC). A higher CGN/NCG motif ratio indicated higher DNA methylation levels, since nucleases responsible for cfDNA cleavage were demonstrated to preferentially cut at methylated CpG sites (15). To infer the methylation status at repetitive *Alu* elements, we downloaded the 'RepeatMasker' track from UCSC´s Genome Browser database (16, 17) and subset the table to only include *Alu* elements. cfDNA fragments with 5'-CGN or 5'-NCG end motifs that overlapped any *Alu* element were extracted and used to calculate per sample CGN/NCG motif ratios.

Supplementary Figure 1

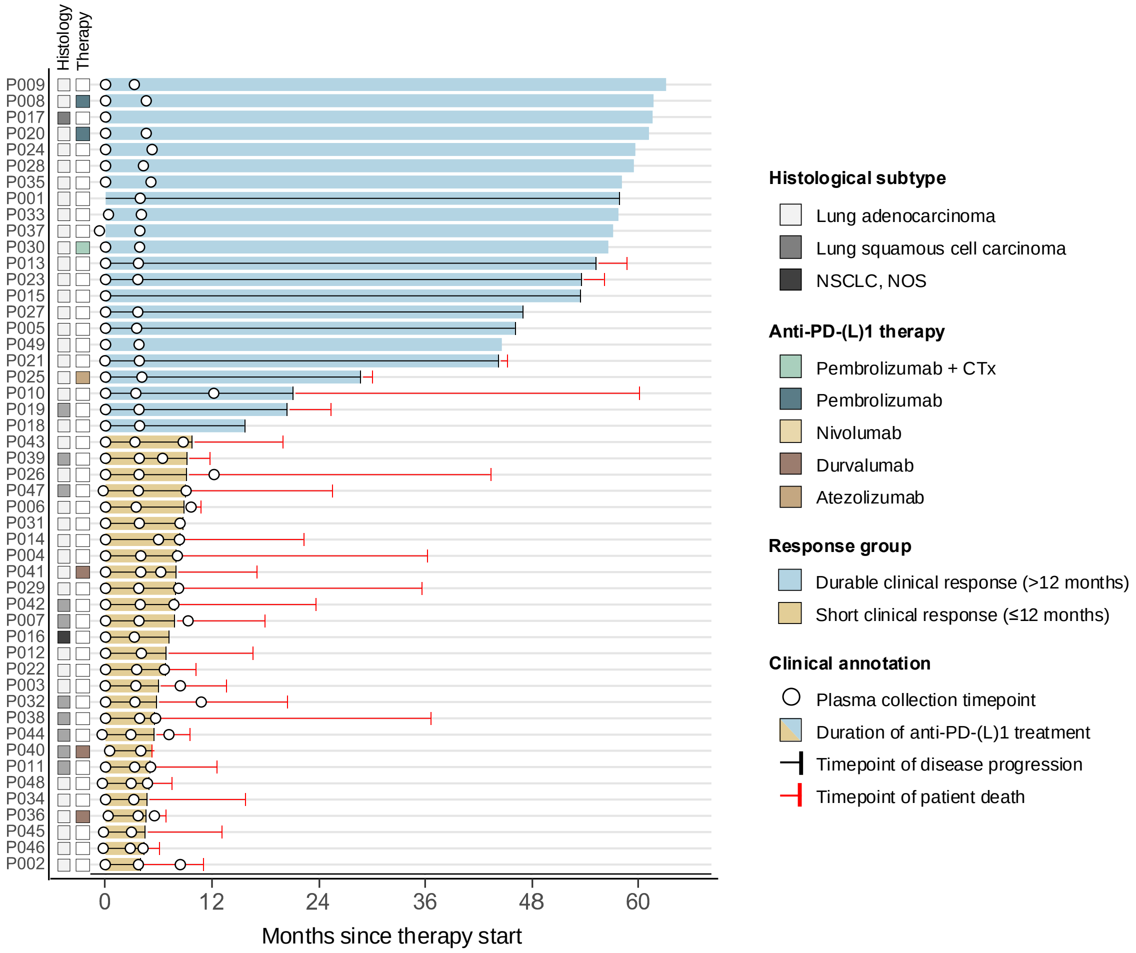

Fig. S1: Swimmer plot illustrating the plasma collection scheme with respect to the start of anti-PD-(L)1 therapy. In addition, the type and duration of therapy as well as the tumor histological subtype is given. Patients are ordered by progression-free survival (PFS) duration and separated into durable and short clinical responders (*i.e.*, PFS >12 months and ≤12 months, respectively). CTx, chemotherapy; NOS, not otherwise specified; PD-L1, programmed death-ligand 1.

Supplementary Figure 2

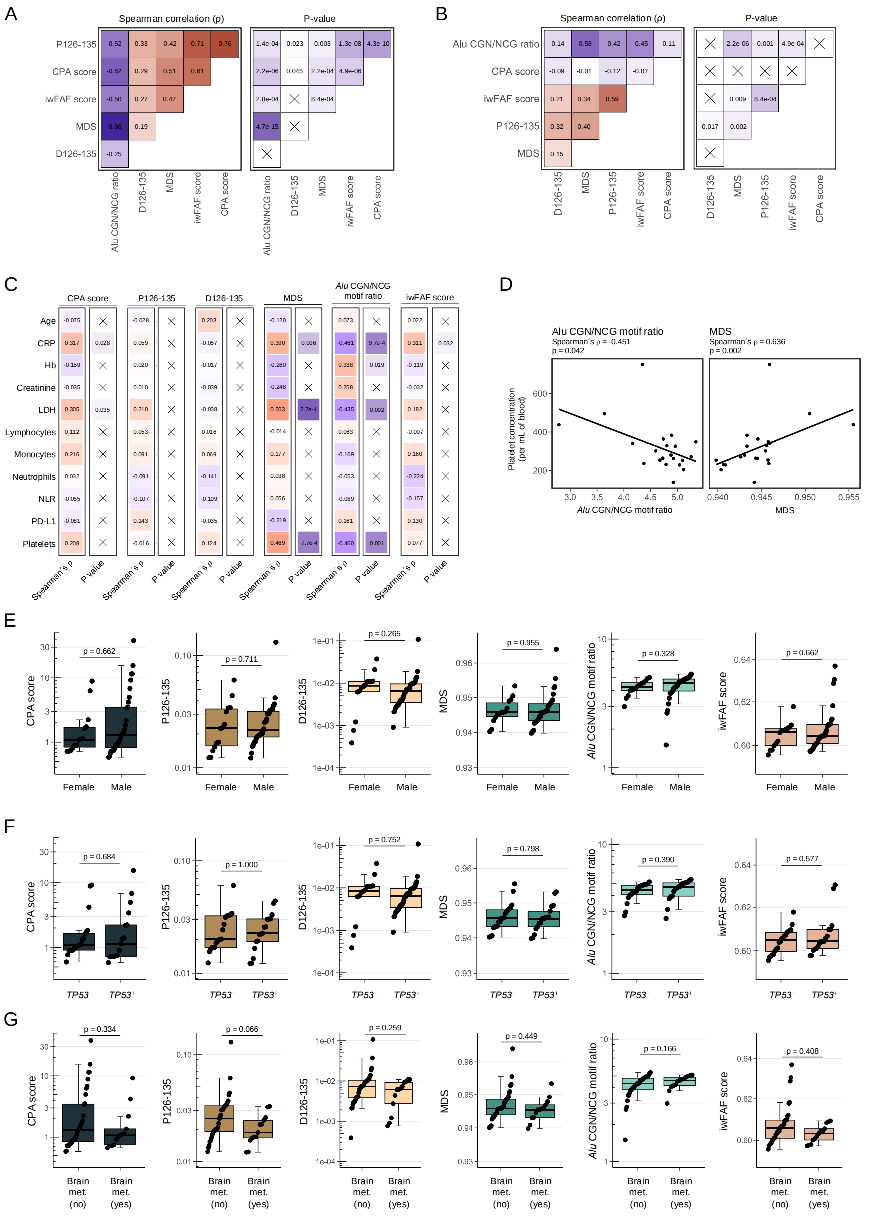

Fig. S2: Correlations between cfDNA biomarkers in pre-treatment NSCLC patient samples (n = 48; A) and healthy donors (n = 57; B). (C) Spearman correlations and corresponding p-values, comparing cfDNA biomarkers to patient age, blood cell counts, and various blood values in all pre-treatment NSCLC patients (n = 48). Box colors indicate the correlation direction (red = positive; blue = negative) and crossed-out boxes show non-significant correlations. (D) Correlations between *Alu* CGN/NCG motif ratios (left) and motif diversity scores (MDS, right) or platelet concentrations in CPA score negative patients (n = 21). cfDNA biomarker comparisons between female (n = 14) and male (n = 35; E), *TP53*-positive (n = 17) and -negative (n = 21; F) patients as well as patients with (n = 13) and without brain metastases (n = 35; G). CPA, copy number abnormality; CRP, C-reactive protein; D126-135, deviation of fragments between 126 and 135-bp from healthy donors; Hb, hemoglobin; iwFAF, information-weighted fraction of aberrant fragments; LDH, lactate dehydrogenase; NLR, neutrophil-to-lymphocyte ratio; P126-135, proportion of fragments between 126 and 135-bp; PD-L1, programmed death-ligand 1.

Supplementary Figure 3

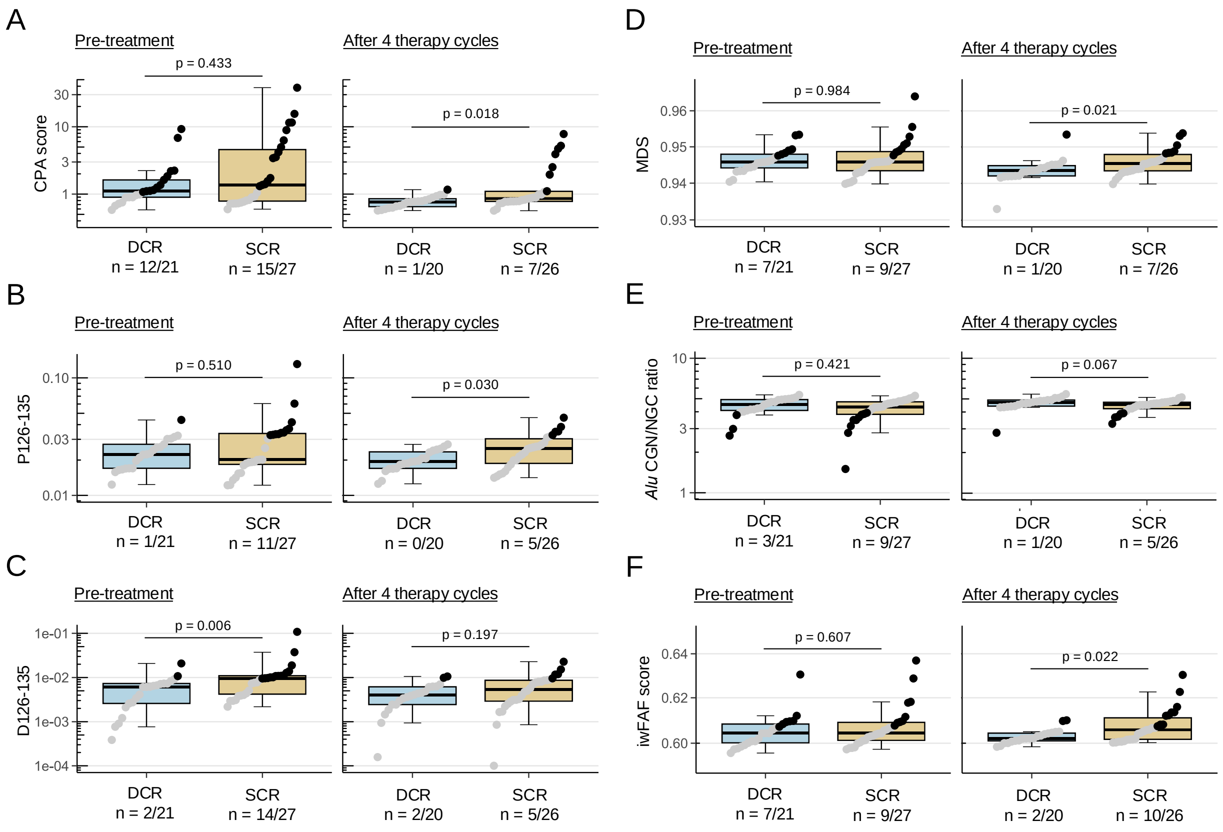

Fig. S3: Comparison between durable (DCR; >12 months) and short clinical responders (SCR; ≤12 months) for CPA scores (A), P126-135 (B), D126-135 (C), MDS (D), *Alu* CGN/NCG ratios (E), and iwFAF scores (F). Patient-specific ctCPA are not illustrated due to their incompatibility for inter-patient comparisons. Dotted lines represent the biomarker´s respective 95% specificity threshold. Samples below this threshold were illustrated in gray. The number of ctDNA⁺ (at 95% specificity) and the total number of samples per group is given below the graphs. Box plots represent median, upper and lower quartile with Tukey Whiskers and statistical significance of independent groups was assess by Mann-Whitney U tests. CPA, copy number abnormality; ctCPA, ctDNA-informed CPA; ctDNA, circulating tumor DNA; D126-135, deviation of fragments between 126 and 135-bp from healthy donors; iwFAF, information-weighted fraction of aberrant fragments; MDS, motif diversity score. P126-135, proportion of fragments between 126 and 135-bp.

Supplementary Figure 4

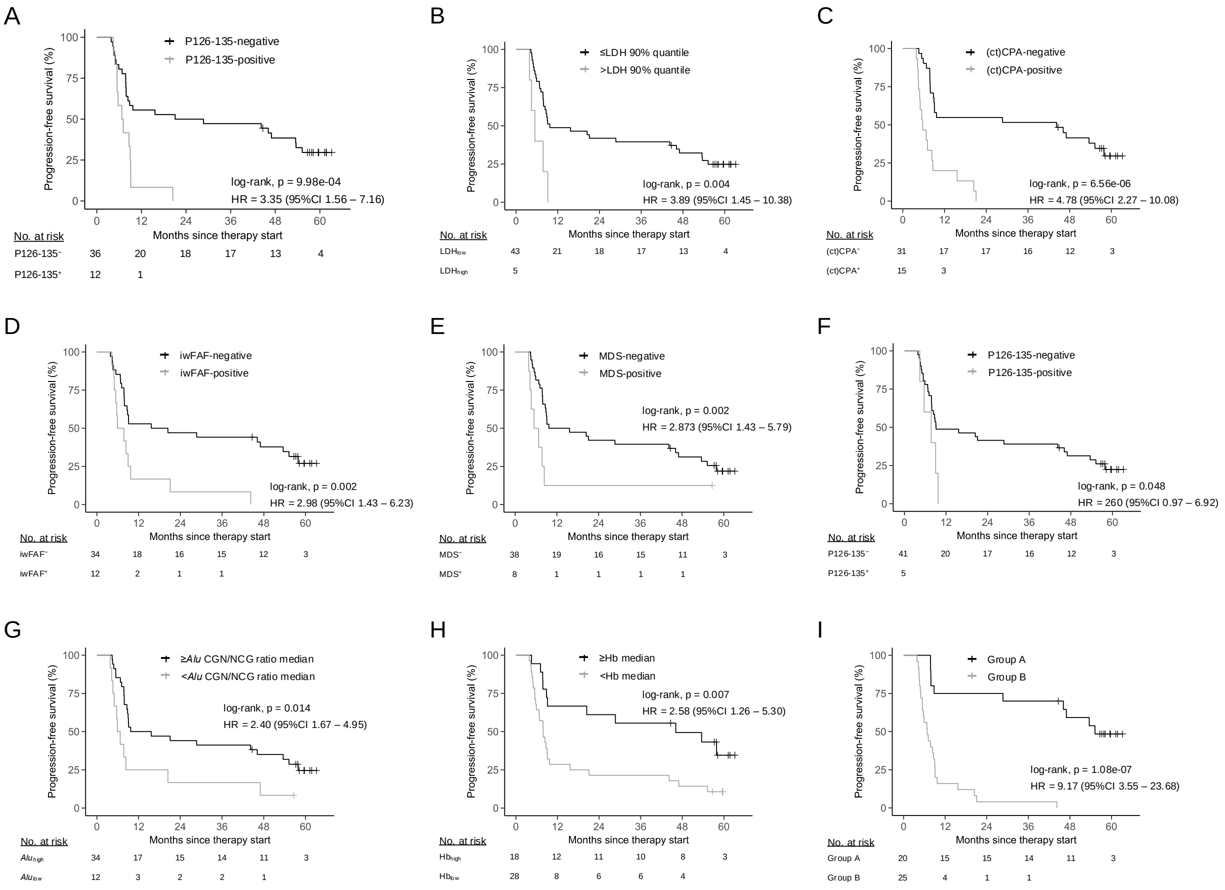

Fig. S4: Associations between progression-free survival (PFS) and pre-treatment P126-135 detectability (at 95% specificity; A), pre-treatment LDH concentrations (B), (ct)CPA score detectability after 4 therapy cycles (C), iwFAF score detectability after 4 therapy cycles (D), MDS detectability after 4 therapy cycles (E), P126-135 detectability after 4 therapy cycles (F), *Alu* CGN/NCG ratios (G) after 4 therapy cycles, and hemoglobin (Hb) molarities after 4 therapy cycles (H). In (I), patients were grouped in Group A when all of the following criteria were met: Undetectable D126-135 levels at baseline and undetectable circulating tumor DNA (ctDNA) after 4 therapy cycles – assessed by iwFAF score and (ct)CPA score detectability. Groups were compared by two-sided log-rank tests and hazard ratios (HR) were calculated *via* univariate Cox proportional hazard models. Quantiles/medians refer to all pre-treatment samples of the presented cohort. ctCPA, ctDNA-informed copy number abnormality; D126-135, deviation of fragments between 126 and 135-bp from healthy donors; iwFAF, information-weighted fraction of aberrant fragments; LDH, lactate dehydrogenase; P126-135, proportion of fragments between 126 and 135-bp; PD-L1, programmed death-ligand 1.

Supplementary Figure 5

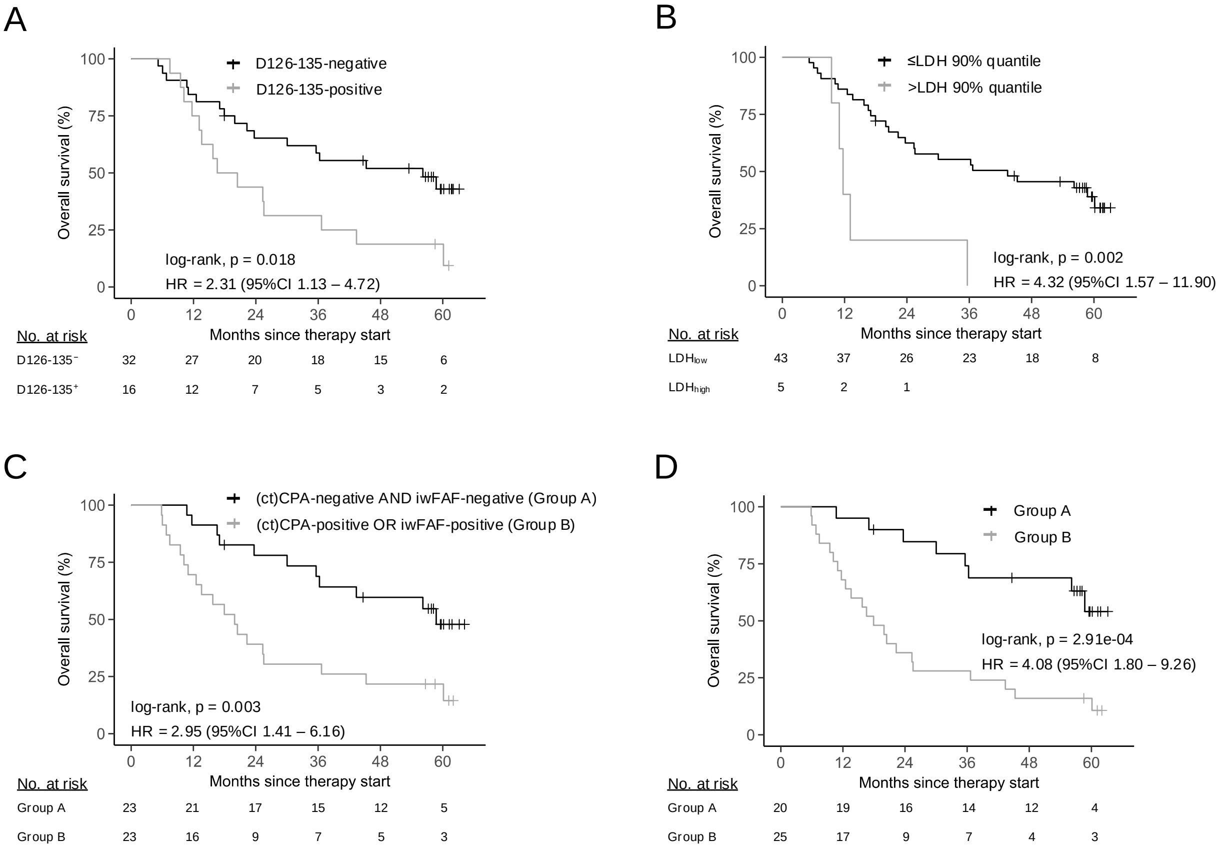

Fig. S5: Overall survival (OS) correlations to pre-treatment detectability (at 95% specificity) of D126-135 (A), pre-treatment LDH concentrations (B), and detectability of (ct)CPA and/or iwFAF scores after 4 therapy cycles. In (E) patients were separated into Group A if all of the following criteria were fulfilled: Undetectable D126-135 at baseline and undetectable (ct)CPA and iwFAF score after 4 therapy cycles. Groups were compared by two-sided log-rank tests and hazard ratios (HR) were calculated *via* univariate Cox proportional hazard models. Quantiles/medians refer to all pre-treatment samples of the presented cohort. ctCPA, ctDNA-informed copy number abnormality; D126-135, deviation of fragments between 126 and 135-bp from healthy donors; iwFAF, information-weighted fraction of aberrant fragments; LDH, lactate dehydrogenase; PD-L1, programmed death-ligand 1.

Supplementary Figure 6

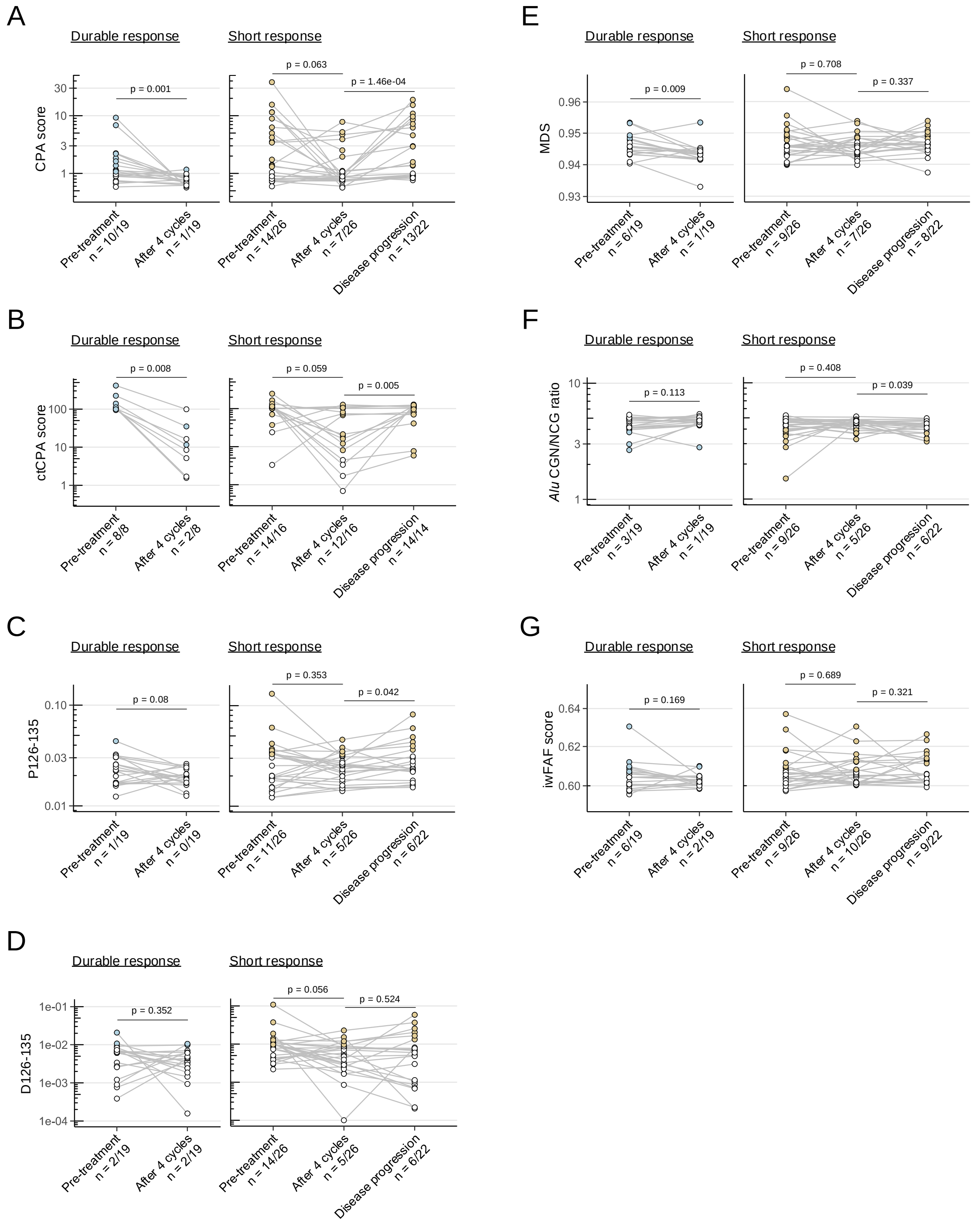

Fig. S6: Biomarker kinetics in durable (DCR; >12 months) and short clinical responder (SCR; ≤12 months) for CPA scores (A), ctCPA scores (B), P126-135 (C), D126-135 (D), MDS (E), *Alu* CGN/NCG ratios (F), and iwFAF scores (G). The dotten line indicates the respective biomarker´s 95% specificity threshold – denoting circulating tumor DNA positivity (ctDNA$^+$) – and filled dots illustrate ctDNA$^+$ samples. For ctCPA in (B), no threshold line is given since ctDNA detectability thresholds vary from patient to patient. The total number of samples per group and the count of ctDNA$^+$ are shown below the graphs. Statistical significance between groups was assessed by Wilcoxon´s paired test.

CPA, copy number abnormality; ctCPA, ctDNA-informed CPA; D126-135, deviation of fragments between 126 and 135-bp from healthy donors; iwFAF, information-weighted fraction of aberrant fragments; MDS, motif diversity score; P126-135; proportion of fragments between 126 and 135-bp.

Supplementary references

1. Martin M. Cutadapt removes adapter sequences from high-throughput sequencing reads. 2011. 2011;17(1):3.
2. Langmead B, Salzberg SL. Fast gapped-read alignment with Bowtie 2. Nature Methods. 2012;9(4):357-9.
3. Li H, Handsaker B, Wysoker A, Fennell T, Ruan J, Homer N, et al. The Sequence Alignment/Map format and SAMtools. Bioinformatics. 2009;25(16):2078-9.
4. Peneder P, Stütz AM, Surdez D, Krumbholz M, Semper S, Chicard M, et al. Multimodal analysis of cell-free DNA whole-genome sequencing for pediatric cancers with low mutational burden. Nat Commun. 2021;12(1):3230.
5. Bonfield JK, Marshall J, Danecek P, Li H, Ohan V, Whitwham A, et al. HTSlib: C library for reading/writing high-throughput sequencing data. Gigascience. 2021;10(2).
6. Moldovan N, van der Pol Y, van den Ende T, Boers D, Verkuijlen S, Creemers A, et al. Multi-modal cell-free DNA genomic and fragmentomic patterns enhance cancer survival and recurrence analysis. Cell Rep Med. 2024;5(1):101349.
7. Raman L, Dheedene A, De Smet M, Van Dorpe J, Menten B. WisecondorX: improved copy number detection for routine shallow whole-genome sequencing. Nucleic Acids Res. 2019;47(4):1605-14.
8. Olshen AB, Venkatraman ES, Lucito R, Wigler M. Circular binary segmentation for the analysis of array-based DNA copy number data. Biostatistics. 2004;5(4):557-72.
9. Raman L, Van der Linden M, Van der Eecken K, Vermaelen K, Demedts I, Surmont V, et al. Shallow whole-genome sequencing of plasma cell-free DNA accurately differentiates small from non-small cell lung carcinoma. Genome Med. 2020;12(1):35.
10. Janke F, Stritzke F, Dvornikovich K, Franke H, Angeles AK, Riediger AL, et al. Early circulating tumor DNA changes predict outcomes in head and neck cancer patients under re-radiotherapy. Int J Cancer. 2024.
11. Johnson WE, Li C, Rabinovic A. Adjusting batch effects in microarray expression data using empirical Bayes methods. Biostatistics. 2007;8(1):118-27.
12. Jiang P, Sun K, Peng W, Cheng SH, Ni M, Yeung PC, et al. Plasma DNA End-Motif Profiling as a Fragmentomic Marker in Cancer, Pregnancy, and Transplantation. Cancer Discovery. 2020;10(5):664-73.
13. Budhraja KK, McDonald BR, Stephens MD, Contente-Cuomo T, Markus H, Farooq M, et al. Genome-wide analysis of aberrant position and sequence of plasma DNA fragment ends in patients with cancer. Sci Transl Med. 2023;15(678):eabm6863.
14. Hinrichs AS, Karolchik D, Baertsch R, Barber GP, Bejerano G, Clawson H, et al. The UCSC Genome Browser Database: update 2006. Nucleic Acids Res. 2006;34(Database issue):D590-8.
15. Zhou Q, Kang G, Jiang P, Qiao R, Lam WKJ, Yu SCY, et al. Epigenetic analysis of cell-free DNA by fragmentomic profiling. Proc Natl Acad Sci U S A. 2022;119(44):e2209852119.
16. Karolchik D, Hinrichs AS, Furey TS, Roskin KM, Sugnet CW, Haussler D, et al. The UCSC Table Browser data retrieval tool. Nucleic Acids Res. 2004;32(Database issue):D493-6.
17. Nassar LR, Barber GP, Benet-Pagès A, Casper J, Clawson H, Diekhans M, et al. The UCSC Genome Browser database: 2023 update. Nucleic Acids Res. 2023;51(D1):D1188-d95.
